# Prognosis of recurrent non-small cell lung cancer following complete resection

**DOI:** 10.3892/ol.2014.1861

**Published:** 2014-02-07

**Authors:** HIDEFUMI SASAKI, AYUMI SUZUKI, TSUTOMU TATEMATSU, MASAYUKI SHITARA, YU HIKOSAKA, KATSUHIRO OKUDA, SATORU MORIYAMA, MOTOKI YANO, YOSHITAKA FUJII

**Affiliations:** Department of Oncology, Immunology and Surgery, Nagoya City University Graduate School of Medical Sciences, Nagoya, Aichi 467-8601, Japan

**Keywords:** EGFR, recurrent, prognosis, tyrosine kinase inhibitor

## Abstract

Prognosis following recurrence subsequent to complete resection of non-small-cell lung cancer (NSCLC) is considered a multifactorial process dependent on clinicopathological, biological and treatment characteristics. Gefitinib was approved for lung cancer treatment in Japan in 2002. The aim of the current study was to quantify the prognostic effects of these characteristics on post-recurrence prognosis. In total, 127 NSCLC patients were analyzed who underwent complete resection and subsequently had recurrent cancer. The correlation between characteristics of the initial and recurrent disease with post-recurrence prognosis was investigated. The factors clearly associated with post-recurrence prognosis using Cox proportional hazards models were age at recurrence (those <65 years of age typically had better prognoses) and interval between initial resection and recurrence (intervals of <1 year accompanied a worse prognosis). Epidermal growth factor receptor (EGFR) mutant patients treated with EGFR tyrosine kinase inhibitors (TKIs), exhibited the longest median survival following recurrence (37.4 months) in the sample. Treatment, particularly EGFR TKIs for recurrent NSCLC, was observed to significantly prolong survival. The results of the study highlight that various treatment modalities according to the clinical background of the patient should be considered in patients with postoperative recurrent NSCLC.

## Introduction

Lung cancer is the leading cause of cancer-associated mortality worldwide. For patients with early-stage (stage I and II) non-small cell lung cancer (NSCLC) and for appropriately selected patients with locally advanced disease (stage IIIA), complete surgical resection is the optimal treatment. Although advancements in early diagnosis and treatment have been made in the hope of improving survival, recurrence remains a major problem. Reported recurrence rates following complete surgical resection range between 30 and 75% (1–6) [~15% for pathological stage (p-stage) I cases (7,8)]. The majority of recurrent tumors are distant and >80% of recurrences occur within the first 2 years after resection.

Several previous studies have evaluated the prognostic factors associated with survival following recurrence of NSCLC. Chemotherapy and radiation therapy are generally accepted treatment options for recurrent NSCLC. Encouraging new treatments [including epidermal growth factor receptor-tyrosine kinase inhibitors (EGFR-TKIs), anaplastic lymphoma kinase inhibitors, pemetrexed and bevacizumab for adenocarcinomas] have benefited specific patients with advanced or recurrent NSCLC (9–15). Identification of activating mutations of EGFR is one of the most important developments in the field of NSCLC. EGFR mutations are present predominantly in females, in never-smokers and in Asian individuals, and are sensitive to EGFR-targeted therapy, including gefitinib (16–18). The response rate to gefitinib is almost 75% in patients with tumors harboring EGFR mutations in Asian clinical trials (9–11,17,18). These advances in post-recurrence therapy may improve overall survival among patients who undergo surgery. The present study investigated the clinicopathological factors influencing post-recurrence survival and the effect of post-recurrence therapy on Japanese NSCLC since 2002.

## Patients and methods

### Patients

The study group included 637 patients with NSCLC: 435 were diagnosed as having adenocarcinomas, 147 had squamous cell carcinomas, 20 had adenosquamous carcinomas and 10 had large-cell carcinomas. All had undergone complete resection at the Department of Surgery, Nagoya City University Hospital (Nagoya, Japan) between 2002 and 2012. Patients who succumbed to disease without identifiable recurrence were excluded from the study. The lung tumors were classified according to the general rules for clinical and pathological diagnosis of lung cancer in Japan (19).

The clinical and pathological characteristics of the 637 lung cancer patients were as follows: 435 cases at stage I, 93 at stage II and 109 at stage III. The mean age was 66.8±9.7 years (range, 22–87 years). Males accounted for 401 cases and 236 were female. Non-smokers numbered 232. Regarding the mutation of EGFR, the majority of samples from these patients had been analyzed previously (16,17,20,21). EGFR mutations were present in 215 cases and 366 were wild type. The remaining 56 were unknown. This study was approved by the ethics committee of Nagoya City University (Nagoya, Japan). Written informed consent was obtained from the patient.

### Post-recurrence survival analysis

Clinical characteristics were retrieved from available clinical records. The following clinicopathological factors were assessed in the post-recurrence analysis: Age, gender, smoking status, pathological stages and histology (adenocarcinoma vs. others). The length of the recurrence-free period was calculated in months between the date of resection and the date of initial recurrence or the date of the last follow-up without recurrence. To calculate the recurrence-free proportion, patients known to have no recurrence at the date of last contact were excluded from this study. The length of post-recurrence survival was measured between the date of initial recurrence and the date on which the patient succumbed to disease or the date on which the patient was last known to be alive.

### Statistical analysis

The overall survival of patients with lung cancers was examined by the Kaplan-Meier methods and differences were examined by the log-rank test. The other clinicopathological characteristics were examined using Student’s t-test and χ^2^ tests as appropriate. Analyses were performed using Statview version 5.0 (Abacus Concepts Inc., Berkeley, CA, USA) or Excel (Microsoft Corporation, Redmond, WA, USA) software and P<0.05 was considered to indicate a statistically significant differences.

## Results

Of the 637 patients, 127 (19.9%) had recurrent diseases, with a median age of 66.6±9.3 years (range, 29–85) at the initial surgery. Median follow-up time for the patients from the initial surgery was 38.3±24.5 months (range, 4–127 months). Median post-recurrence survival time for these patients was 21.9±18.4 months (range, 1–98 months). The 1- and 2-year post-recurrence survival proportions were 69.8 and 44.4%, respectively. The recurrence rate was 12.6% in stage I cases, 37.6% in stage II and 33.9% in stage III.

**Figure 1:**
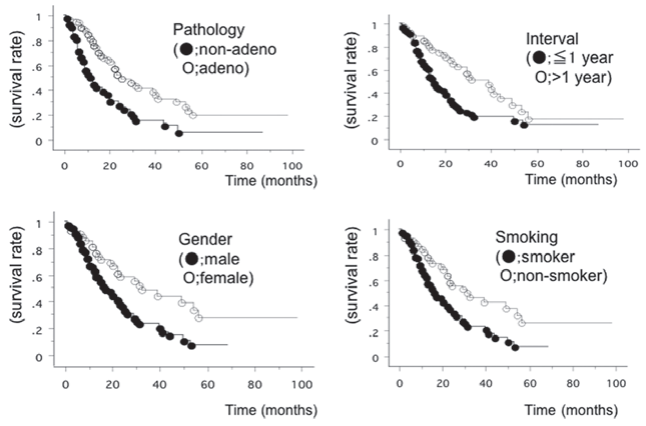
Survival curve for the top 4 prognostic factors.

As shown in Table I, univariate and multivariate analyses of recurrence, according to the clinicopathological characteristics of NSCLC patients, were performed. Univariate analysis identified 5 significant risk factors: Male gender (P=0.0024), a history of smoking (P=0.0026), non-adenocarcinoma histology (P=0.0002), an age of ≥65 years old at recurrence (P=0.0060) and a recurrence interval of ≤1 year (P=0.0016) (Fig. 1). Multivariate analysis demonstrated that below 65 years old [hazard ratio (HR), 2.299; 95% confidence interval (CI), 1.374–3.846; P=0.0015) and a recurrence interval of <1 year (HR, 2.119; 95% CI, 1.296–3.464; P=0.027) were statistically significant predictors of shorter survival. EGFR mutation itself was not a prognostic factor.

**Figure 2:**
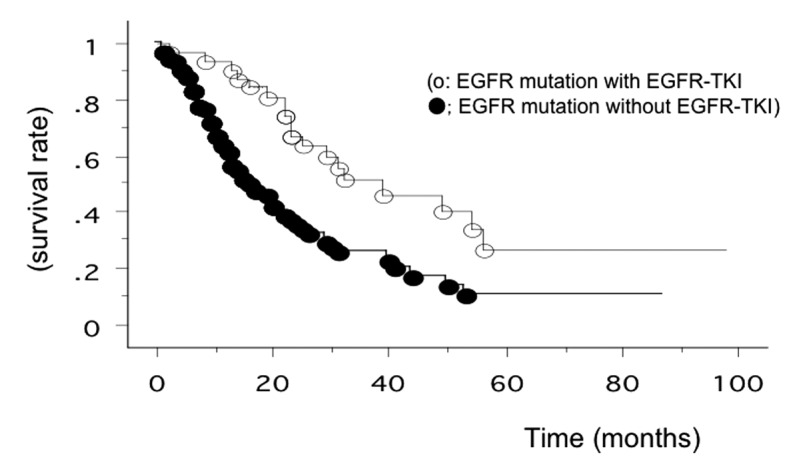
Subgroup analysis revealed that the overall survival of the 29 EGFR-mutant patients treated with EGFR-TKI was 37.4 months (range, 2–98 months after recurrence) and that of other patients was 22.5 months. The median survival of the EGFR-TKI-treated patients with EGFR mutation was the longest in this analysis. EGFR, epidermal growth factor receptor; TKI, tyrosine kinase inhibitor.

Initial recurrence sites are shown in Table I and post-recurrent treatment is shown in Table II. Liver metastases were associated with a trend towards poorer prognosis (P=0.091). However, none of the involved organs, including the bones and brain were significant prognostic factors. Post-recurrence treatment was administered in 114 patients (89.8%), including chemotherapy for 99 patients and radiotherapy for 82. Univariate analysis revealed the administration of chemotherapy, but not radiotherapy, to be a prognostic factor. EGFR-TKIs were used in 39 cases and such therapy was revealed to be a marginal prognostic factor by univariate analysis. However, these were not significant prognostic factors according to multivariate analysis. Among the 39 patients treated with EGFR-TKIs, 29 had EGFR mutations. Subgroup analysis placed the overall survival of the 29 EGFR-mutant patients treated with EGFR-TKIs at 37.4 months (range, 2–98 months after recurrence), while that of other patients was 22.5 months. The overall survival of EGFR-TKI-treated patients with EGFR mutation was the longest in the present study (Fig. 2).

## Discussion

In a previous study, Yoshino *et al* reported that post-recurrence survival of NSCLC patients who underwent complete resection was significantly affected by gender, age at recurrence, p-stage, pulmonary metastasis and recurrence interval (3). In this study, further analysis was conducted with special reference to organ and therapy. Therefore, patients with recurrence were selected between 2002 and 2012 when gefitinib had become a key drug for NSCLC. The results of the present study are similar in part.

The prognostic effect of initial lung cancer stage on survival following recurrent cancer has been investigated. Advanced stages of the initially resected NSCLC have been shown to be associated with increased rates of recurrence and shortened recurrence-free intervals (3,22–24), suggesting the utility of p-stage as a marker of tumor aggressiveness or occult disease at resection. The association of stage with post-recurrence survival has been demonstrated previously, with advanced stages accompanying a 30–90% increased risk of mortality (3,22,23). Post-operative recurrence is understood as the reappearance of latent cancer cells known as micrometastases. Therefore, the positive correlation between advanced p-stage and high recurrence rate or a short disease-free interval is well understood. However, in the present study p-stage was not a prognostic factor. Appropriately staging and chemotherapy, including EGFR TKI therapy, may overcome the initial pathological stages. However, disease-free interval was a prognostic factor. Walsh *et al* (23) characterized disease-free interval as an “indirect measure of a patient’s tumor biology and aggressiveness”. Thus, longer disease-free interval has been reported to be associated with prolonged survival following recurrence in several studies (24–27).

Major advances in NSCLC treatment have resulted from the understanding of the molecular biology of the disease, the development of molecule-targeting agents and the identification of biomarkers for targeted treatment. Since 2002, gefitinib therapy has been approved for the treatment of inoperable or recurrent NSCLC in Japan, hence the focus of the present study on cases subsequent to 2002. EGFR-TKIs have been proven to improve the survival of certain advanced NSCLC patients (28–30), with the overall benefit being determined primarily by the EGFR mutation subgroup (9–11,16,17,31). EGFR-TKIs have also improved endurance and health-related quality of life compared with platinum-based doublet chemotherapy (9–11). EGFR-TKIs are therefore good candidates for first-line post-recurrence treatment in resected adenocarcinoma patients with distant metastases, but only in those with EGFR mutations (11,28).

There are several limitations in the present study. This study is retrospective and bias may exist. Patient selection bias regarding post-recurrence treatment was unavoidable. Curative intent therapy or systematic treatment is difficult to perform in patients with poor performance status and therefore younger patients had better prognoses. In addition, complete follow-up was not available for all eligible patients. One challenge for the future is to create systematic treatment strategies for recurrent NSCLC according to the individual patient’s recurrent disease characteristics, including the initial recurrence site, recurrence-free interval and original tumor characteristics.

## Figures and Tables

**Table I:** Clinicopathological data of 127 lung cancer patients.

	Prognostic analysis			
	
	Univariate			Multivariate
		
Factors	Patients, n	MST, months	P-value	HR (P-value)
Age, years			0.0060	2.299 (0.0015)
<65	41	34.2		
≥65	86	23.2		
Smoking history			0.0026	1.378 (0.4548)
Non-smoker	81	22.2		
Smoker	46	34.0		
Pathological subtypes			0.0002	1.541 (0.0752)
Adenocarcinoma	86	31.6		
Non-adenocarcinoma	41	17.9		
Gender			0.0024	1.201 (0.6771)
Male	83	22.3		
Female	44	34.5		
Recurrence interval, years			0.0016	2.119 (0.0027)
<1	70	21.4		
>1	57	34.1		
Brain metastasis			0.2596	
Yes	18	14.5		
No	109	27.7		
Bone metastasis			0.4111	
Yes	28	24.1		
No	99	27.8		
Liver metastasis			0.0910	
Yes	4	14.5		
No	123	24.8		

MST, mean survival time; HR, hazard ratio.

**Table II:** Treatment of 127 patients with recurrent lung cancers.

		Prognosis		
			
Treatment	Samples, n	MST, months	Two-year survival, n (%)	P-value
Chemotherapy				0.0007
Yes	99	29.6	37 (49.5)	
No	28	16.3	4 (23.2)	
EGFR TKIs				0.0315
Yes	39	32.8	19 (55.0)	
No	88	23.6	23 (39.2)	
EGFR mutations				0.0563
Yes	40	31.6	20 (50.0)	
No	87	24.2	22 (37.8)	
Radiotherapy				0.3832
Yes	82	25.4	27 (42.8)	
No	45	29.8	15 (47.8)	

MST, mean survival time; EGFR, epidermal growth factor receptor; TKI, tyrosine kinase inhibitor.
